# Vessel identification based on automatic hull inscriptions recognition

**DOI:** 10.1371/journal.pone.0270575

**Published:** 2022-07-19

**Authors:** Natalia Wawrzyniak, Tomasz Hyla, Izabela Bodus-Olkowska

**Affiliations:** 1 Department of Navigation, Maritime University of Szczecin, Szczecin, Poland; 2 Department of Computer Science, West Pomeranian University of Technology, Szczecin, Poland; 3 Marine Technology Ltd., Gdynia, Poland; University of Engineering & Technology, Taxila, PAKISTAN

## Abstract

The identification of ships plays a crucial role in security and managing vessel traffic for ports and onshore facilities. Existing video monitoring systems help visually identify a vessel where other systems are not present or sufficient. Readable vessel plates and hull inscriptions of detected ships in the video stream allow using text location and recognition methods to obtain ships’ identification names or numbers. The obtained information can be then matched with available ship registers. The automation of the process has met many challenges related to the often-low quality of available video streams, heterogeneous regulations on the marking of ships, and the specifics of natural scene text recognition, such as quickly alternating imaging conditions or the interference of the background. The main contribution of this research is a method that can identify any type of vessel in an image that has visible inscriptions (name, registration number) placed on the hull and must be registered in a public registry. The proposed method works with low-quality images with inscriptions placed under different angles and different, readable sizes. Our method recognised 91% of vessels from our test dataset. Obtained identification times have not exceeded 1s. The quality and efficiency of the proposed solution indicate that it is suitable for practical implementation in onshore monitoring systems.

## Introduction

The identification of ships is crucial for maintaining safety in waterway traffic navigation and is of great importance to ports and waterside infrastructure. Ship identification means vessel detecting, recognising and matching the existing data on units capable of navigating specified areas. There are several ways to identify a vessel, depending on specific needs: dedicated Automatic Identification System (AIS) transponders [1], radar systems [2] for tracking and tracing, Long-Range Identification and Tracking (LRIT) that uses voice communication to confirm identity [3], etc. However, visual confirmation is vital in many cases, and onshore video surveillance systems play that part. In most cases, visual identification is made manually by an observer in a monitoring centre. However, some ports or Vessel Traffic Service (VTS) centres automate the process, especially ship detection and tracking. However, a few tasks need to be performed to recognise and identify a ship from a video stream automatically. First, each ship must have at least one identifier assigned and present in one of the existing ship registries. The marking of the ship needs to include identifiers that are readable from where surveillance cameras are located. The system must detect any vessel that enters a monitored area (the problem of the detection and tracking of ships from video streams was discussed in [4–7]). A text recognition solution must locate and recognise the hull inscription on that vessel and check it against a list of authorised ships in the monitored area.

Multiple systems assign ships identifiers like International Radio Call Sign (IRCS), Maritime mobile service identity (MMSI), or European Vessel Identification (ENI) for inland ships [8]. Some of them assign a Unique Vessel Identifier (UNI) that is never reused and stays with a vessel for its lifetime, such as an IMO number. However, ships’ inscriptions and vessel plates depend on whether the unit is subject to SOLAS [9] convention or some local registration regulations. If the vessel is under SOLAS recommendations, this determines inscriptions to expect and where their placement is supposed to be. All SOLAS ships have an IMO number, and their data are stored in a database under International Maritime Organization (IMO) administration. This rule applies to all large marine seagoing commercial and passenger ships worldwide. Such ships’ markings are relatively easy to recognise by automatic systems and check against the database. Large inland vessels, such as barges and push or towing vessels, have their own rules regarding inscriptions and unit markings, and Inland Hull Databases cover their data. However, not many regulations on inscriptions apply to pleasure crafts like small boats, small yachts, or rowing boats. Moreover, they can be registered in respective local authorities’ registries and have the ship name placed anywhere on the hull, painted in different fonts and colours.

**Fig 1:**
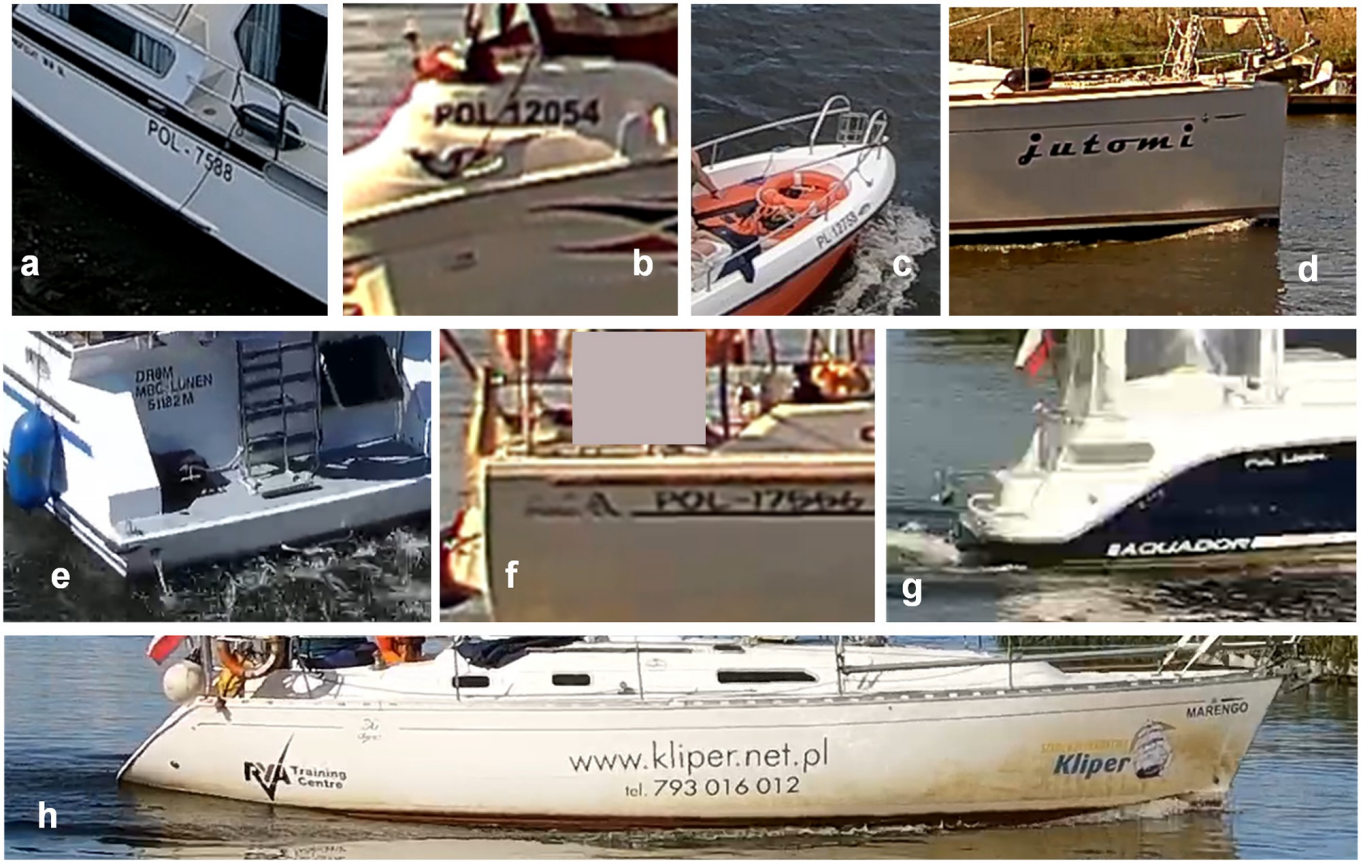
Examples of problematic hull inscriptions. Sample pictures taken in Szczecin Waterway Node area in Poland (own recordings).

In general, detecting and recognising text in a natural scene involves many known challenges related to either poor and quickly alternating imaging conditions (e.g., weather and daylight) or the interference of the background (unpredicted objects or occlusions from other things [10]. On the water, many factors hinder this task. Lighting conditions are additionally demanding due to sun reflection from the waves. The placement of monitoring cameras (often over or under a bridge in inland systems) does not help with the high contrast, and scenes can be half over or underexposed. High humidity causes air transparency to decrease, which blurs the image. Another group of issues are caused by nonhomogeneous ships markings and weak regulations on hull inscriptions and their visibility. Some vessel markings use fancy fonts (Fig 1d) or unusual name placement (Fig 1g) or cause the plate number to blend in with other graphics (Fig 1f). Also, there are more inscriptions besides a name and a registration number/vessel identifier (Fig 1h). In addition, small occlusions caused by mooring ropes or fenders (Fig 1a and 1b) make successful plate recognition a challenge.

Additionally, camera position and movement caused by high winds can be problematic in a coastal environment. Another challenge is the low resolution of monitoring cameras and the signal compression in systems’ data transfer, which significantly impacts the analysed images. This causes the quality of the baseline image to be low. The images contain visible compression artefacts and inscriptions of 10 to 20 pixels in height, making it difficult to read the inscriptions correctly.

**Fig 2:**
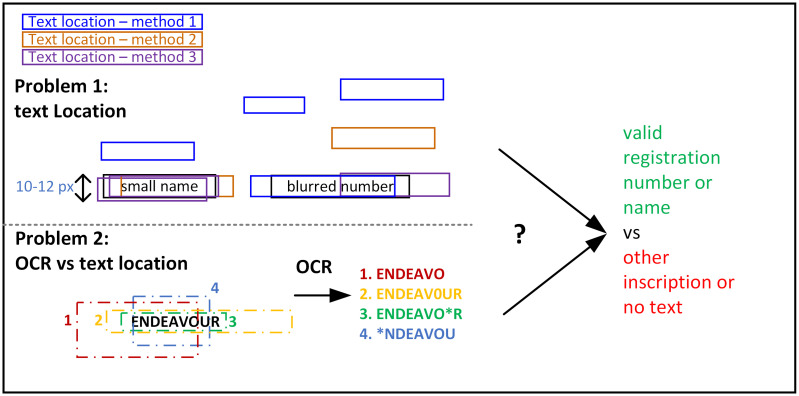
Illustration of the inscription recognition problem.

Two specific problems (Fig 2) need to be addressed in such cases. First, different text localisation algorithms might return slightly different inscription regions or completely erroneous results. Second, the optical recognition quality often depends on how the inscription is cut out from the scene, especially using algorithms based on deep learning. A larger or smaller margin might produce better or worse results (with only a few pixels’ difference). It is impossible to verify which margin is better before the optical recognition process. Moreover, Fig 2 does not present inclined inscriptions that must be rotated. This also causes quality problems. Additionally, the inscriptions read are not 100% correct in many cases. The problem is how to use a set of partially correct and incorrect text strings to find a proper match in a ship registry.

### Related works

Text recognition in a natural scene is a research topic that has been explored for at least 30 years. Its main achievements from before the deep learning era have been described in detail by [5]. However, the rapid development of methods based on convolutional neural networks (CNN) has allowed for significant advances that were brought together in a survey [11]. In this extensive overview, Long et al. described recent developments in text detection and recognition research and pointed out areas where further research is needed. What is noticeable is that the use of CNN networks has allowed researchers to approach the problem of text recognition from many perspectives.

A common way is to divide the process of text recognition into two tasks. The first is text localisation in the image, and the second is its recognition by some form of Optical Character Recognition (OCR) method. The problem of recognising text in previously localised areas is commonly solved by an OCR tool such as OCR4all [12] for handwriting or Calamari [13] based on text lines. There is ongoing advancement in this area of research, mainly using deep learning tools and adjustments [14]. However, the most used and documented OCR is Tesseract [15, 16].

However, the more significant challenge remains the problem of the localisation of text in the image. It can be solved by one of three standard groups of approaches. The first is by using Connected Components Analysis (CCA) methods [17], which are built on chosen scene text characteristics (stroke, colour, etc.). The most often used are Stroke Width Transform (SWT) [18] and Maximally Stable External Regions (MSER) [19–21]. The second group are detection-based methods (either proposal- or regression-based [22, 23]) that use Convolutional Neural Networks (CNN) and can detect multi-oriented scene text. One of these methods is EAST [24], which detects words from quadrilateral shapes and arbitrary orientations. The third group are CNN methods that work on a pixel level to distinguish text from nontext regions. These methods vary in determining these regions, e.g., by specific text segments [25] or corner localisations [26]. Recent developments in this field are up-and-coming, e.g., a method that uses multiscale convolution features to learn the text and offset masks [27]. Other exciting methods that address problems present in vessel image analysis are presented in [28] and [29], which address the issue of irregular text on many leisure craft units [30]. Some approaches combine different methods to achieve specific results [31]. Qin et al. presented a system in which semantic segmentation [32] and the multibox method were processed parallel, mutually correcting the results.

Research tailored for ship detection and recognition from camera images is also present in the literature. Zhang et al. [33] proposed a method that uses CNN to locate ships and detect plate text lines with a Fully Convolutional Network (FCN). It compares the results with the popular EAST method. The recognition and identification part uses available AIS information, so it is designed to work with large commercial maritime units and recognise Chinese characters.

Huang et al. proposed an end-to-end method for vessel plate number recognition [34], which does not treat the detection and identification of vessels plates as separate tasks but is intended to work with naval ship images that show differently arranged vessel numbers.

### Motivation and contribution

This paper is a part of ongoing research in the Ship Recognition (SHREC) project [35], which concerns the automatic recognition and identification of unconventional vessels. The identification method is the last step in the automatic vessel identification and classification process.

The main contribution of this research is a method that can identify any registered vessel visible in an image, which has readable hull inscriptions (name, registration number or identifier) and whose data are available in a registry. The proposed method is designed to work with low-quality images of onshore surveillance systems that monitor vessels of different types. Existing nonhomogeneous regulations, especially for pleasure crafts markings, allow hull inscriptions with low foreground-background contrast under different angles and sizes. The paper contains results of experiments using a dataset containing images of marine and inland vessels obtained from various surveillance cameras placed in Vessel Traffic Services (VTS) and River Information Services (RIS) areas. In the case of many recognised text strings for a single vessel, all of them are analysed and checked against a registry. The one with the best match is chosen as the correct one. If e.g., the name and plate number have the same match quality, the registration number or identifier takes priority.

### Paper organisation

The rest of this paper is organised as follows. Section 2 contains the description of a novel Vessel Identification Scheme. Section 3 presents our test environment, test application, and experimental datasets. The final section discusses the experimental results and provides conclusions concerning the practical implementation of the proposed method.

## Materials and methods

The proposed vessel identification scheme was created using a design science methodology. We first identified the problem of reading ship identification inscriptions for images derived from poor quality video streams. We encountered this problem while developing the ship identification mechanism. We prepared test collections of caption images that we obtained from video streams from surveillance cameras. It became evident that the standard approach, i.e., using only one text localisation and OCR method, cannot provide sufficient quality. It is necessary to propose a more complex solution that uses an auxiliary register of ship identification data. We aimed to obtain a method that recognises as many subtitles as possible (in the percentage of recognised subtitles from the dataset) and that at the same time does not execute longer than 1 second on a test machine, as it would then be useless in practical implementation. The solution presented in the following is the final version of the method, which we obtained by checking and testing various possible ideas on the dataset.

### Informative description

The vessel identification scheme (VIS) comprises four major components: a scene text detection algorithm, optical character recognition engine, text parser, and text matcher. The method as an input accepts an image and outputs a match result, i.e., information about a ship identified in one of the ship registries based on a text read from the input image. The method can use several scene text detection algorithms to improve accuracy, and it is possible to use one of the OCR engines. The method is designed to work on low-quality (highly compressed) streams from surveillance cameras, where inscriptions often have a height of around 10 to 20 pixels and have different fonts. The method can work in different modes that use one to three scene detection algorithms. More than one detection algorithm is sometimes required as low-quality text might not be detected by one method but can still be detected by another.

**Fig 3:**
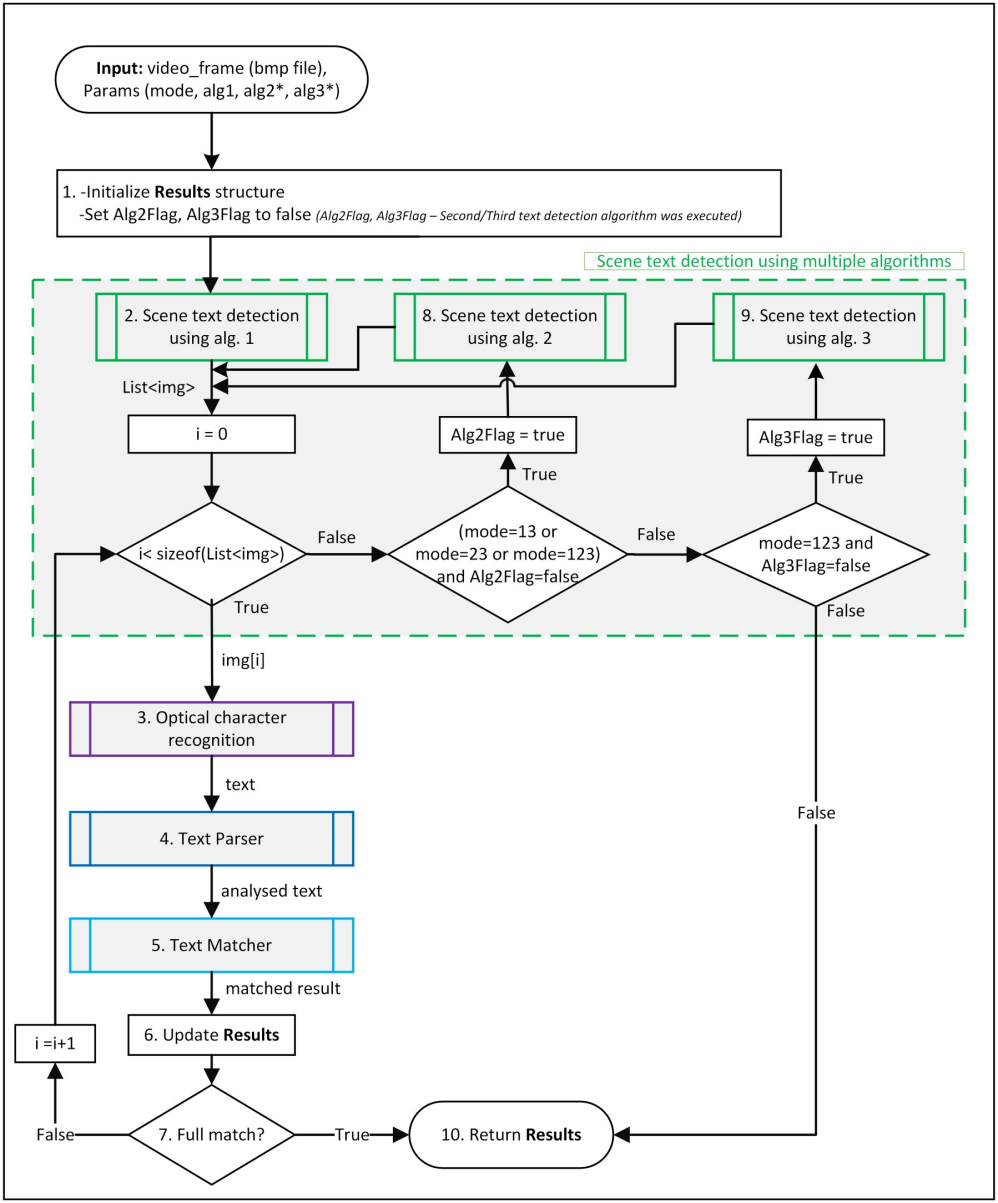
General scheme of vessel identification scheme.

The method works in several steps (compare with Fig 3). In the first preliminary step, the match result that stores the best match result is set to “None.” In the second step, text areas are identified in the image and then cut out, resulting in a list of smaller images with possible inscriptions. Next, every image from the list is sent one by one to the OCR engine (step 3), the output is analysed by the parser (step 4), and finally, the matching algorithm tries to find a vessel in the registries that have inscriptions like the one that was found (step 5). The results can be exact (name or registration number one-to-one match); in such a case, a complete match is returned, and the method ends (steps 6 and 7). Otherwise, the result with high probability is stored as the best match result, and the method continues with the following image. When all possible text areas have been analysed and a full match is not found, the method, depending on the mode of operation, goes to step 8 or 9 and repeats steps 3–7. The method also returns “multiple” results when several high probability matches of two different ships are found.

### Vessel identification scheme

The Vessel Identification Scheme is described in this section, starting with input and output and ending with the detailed steps (Fig 3). The input to the method is an image (a part of the video frame with the detected moving vessel. The parameters are mode of operation: **mode 1** (uses a scene text detection CCA method [15]), **mode 2** (uses MSER method [17]), **mode 3** (uses EAST Detector [22]), and **combined modes**: **12**, **13**, **23**, and **123** that use two or three scene text detection algorithms.

The method outputs a match result structure containing identified inscription and match type. The following match types are possible:

- full—an exact match with the vessel name or registration number was found, i.e., the text read was in the identification data in the register (vessel name or registration number read).
- high—a match with one error was found, i.e., the text read differed by only one character from one entry in the vessel register.
- low—a match with two errors was found, i.e., the text read differed by two characters from one entry in the vessel register.
- multiple—two or more matches, high or low, were found, i.e., a situation in which the method found several captions on an image that were matched with one or two errors. This case is not an exact identification.
- none—no vessel was identified.

By default, the following external algorithms are used, depending on the mode of operations:

- Mode 1—scene text detection based on the CCA method [15].
- Mode 2—scene text detection based on the MSER method [17].
- Mode 3—scene text detection based on the EAST Detector [22].

Additionally, the method uses Tesseract OCR [13] as the OCR engine.

The method has the following steps:

1. Initialize a **global match structure**. In this structure, match results are stored until a full match is found or the method ends.
2. Find probable text areas using the **DetectAreas** algorithm with **input param** equal to 1 in modes 1, **2** in modes 2 and 12, or **3** in modes 3, 13, 23, 123).
3. Use the **MatchVessel** algorithm to obtain a local match result for text areas from the previous step.
4. Update the **global match structure**:
  4.1 If the local match is better, assign the local match to the global match structure.
  4.2 If the local match is a **full match**, then **return the result** and end the method.
  4.3 If the local match has the same match level as the global match, but different vessels were recognised, change the result to **multiple matches** and save information about both vessels.
5. If mode is equal to 13, 23, 12, or 123, then:
  5.1 Find probable text areas using the **DetectAreas** algorithm with **input_param** equal to **1** in modes 13 and 12 or 2 in modes 23 and 123.
  5.2 Repeat steps 3 and 4.
6. If mode = 123, then:
  6.1 Find probable text areas using the **DetectAreas** algorithm with **input param** = 1.
  6.2 Repeat steps 3 and 4.
7. Return the global match structure. If the method reaches this step, it means that no ship was identified, and the returned match type is equal to “none.”

### Algorithm DetectAreas (input param)

1. Find text areas (described by rotated rectangles):
  1.1 If input param = 1 uses Alg. 1, i.e., Connected Component Analysis.
  1.2 If input param = 2 use Alg. 2, i.e., Maximally Stable Extremal Regions.
  1.3 If input param = 3 use Alg. 3, i.e., EAST Detector.
2. Filter out any rotated rectangle RR that:
  2.1 Has an angle >45° or <45°.
  2.2 Has RR width < RR height.
  2.3 Has (RR Width / RR Height) < 2.0.
  2.4 Has (RR Height < 10 or RR Width < 20); allow a minimum of two signs larger than 10 pixels.
  2.5 Has (RR Width > 600 or RR Height > 100); two large rectangles are not titles.
3. If input param = 1, then:
  3.1 Deskew and crop the text images from the input image using rotated rectangles.
4. If input param = 2, then:
  4.1 Deskew and crop the text images from the input image using rotated rectangles (use offset = 4 to allow for additional pixels on borders).
5. If input param = 3, then:
  5.1 for i = 1 to 3 (3 times)
    5.1.1 Deskew and crop the text images from the input image using rotated rectangles (*height*+2*i, *width*+2*i); (because the EAST detector on low-quality, small text sometimes does not output accurate results, the OCR engine works better with different offsets, depending on the input image; more text can be detected in contrast to using only one text image when three versions with different borders are used).

### Algorithm MatchVessel (input List<image>)

1. For each image from List<image>) (executed in parallel)
  1.1 Prepare for OCR. Enhance image contrast and equalise the histogram. (This step was disabled during tests as it reduced the detection quality.)
  1.2 Use the OCR engine to get texts from the image.
2. Parse all texts from step 1, for each text string:
  2.1 Remove null strings or strings with only white spaces.
  2.2 Remove spaces.
  2.3 Split text on Line Feed character (LF,’\n’);
  2.4 For each string from step 2.3 (in parallel):
    2.4.1 Detect Country Code.
    2.4.2 Detect “SAR” ships.
    2.4.3 Remove short strings length less equal to 3.
    2.4.4 Insert asterisk signs to all characters except for letters and digits and ‘-‘; (all characters present in vessels inscriptions).
    2.4.5 When a string contains more than 50% of asterisks, discard it.
    2.4.6 Remove asterisks at the beginning and end of the string.
    2.4.7 Detect known registration numbers with set patterns (e.g., IMO number, Polish Sea Yacht—POL). For registration numbers with a check digit(s) like IMO number, **return the full match** when such a number is detected.
    2.4.8 Remove ‘-‘.
    2.4.9 If one of the strings was matched as a known registration number, discard the rest of the strings.
    2.4.10 Discard the string if it is a duplicate (including the previous text detection method). All texts detected for the image are stored globally until the end of the method execution.
    2.4.11 Create a list of strings from the previous step.
3. Match vessel to the name and registration number from the registries:
  3.1 Initialize the **match** result as **none**.
  3.2 For each string from step 2:
    3.2.1 Normalize the string, change the letters to uppercase, change “I” to “L”. (Small I (1) and l (L) are almost the same).
    3.2.2 Look for equal 1:1 string in the registries; when found, return a **full match** as the **match result** and end the algorithm.
    3.2.3 Look for matches with one error; when found, set the **local match result** to **high**; the following errors are allowed:
      3.2.3.1 a missing sign at the beginning or end of the string.
      3.2.3.2 the string has the same length as a string from the registry, but one sign is different.
      3.2.3.3 the detected string is missing a sign in the middle.
      3.2.3.4 an additional sign is present at the beginning or end of the string.
    3.2.4 Look for matches with a maximum of two errors; when found, set the **local match result** to **low**; the following errors are allowed:
      3.2.4.1 two errors; the string has the same length as a string from the registry; the length of the string is equal to or greater than 6.
      3.2.4.2 two additional signs at the beginning or end are present; the length is equal or greater than 7.
      3.2.4.3 fix known prefixes (e.g., POL prefix); then, one error can be present.
      3.2.4.4 a maximum of two additional signs are present in any place; the length is equal to or greater than 7.
    3.2.5 Update result:
      3.2.5.1.1 When the local match result is better than the current **match result**, set the match result to the **local match result**.
      3.2.5.1.2 When the local match result is of the same level as the match result, but different vessels were recognised, change **match result** to **multiple**.
  3.3 Return the **match result**.

## Results

### Test environment

The video streams with ship images were collected by authors at the Szczecin Waterway Node in Poland, where inland waters monitored by the RIS centre of Lower Oder River meet the port area of Szczecin, monitored by marine VTS. The recordings were made at the water areas being in jurisdiction of their authorities and with their direct knowledge and support confirmed for the project by letters of support (Marine Office in Szczecin for Marine Technology Ltd from the 17^th^ of March 2017) and collaboration agreement (between Marine Technology Ltd and Inland Navigation Office in Szczecin from the 24^th^ of April 2020). Navigational participants in these areas are well aware of existing surveillance systems. Some of the camera views are publicly presented online. Under the cooperation agreement we have also gained direct access to the RIS video streams. The obtained video streams and pictures were analysed only to detect and identify navigational units.

**Fig 4:**
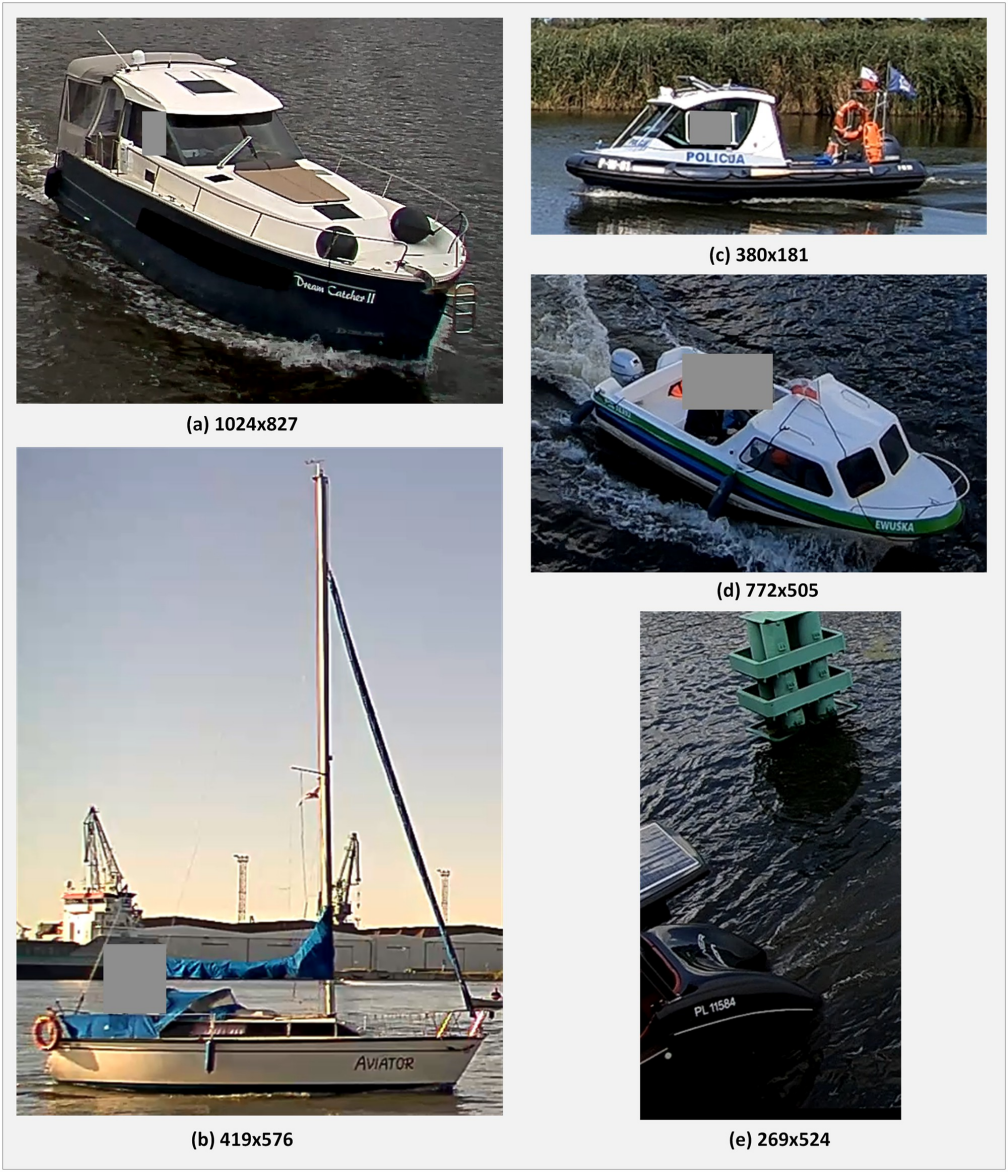
Example images from dataset A with the size in pixels configured to work in the following settings: Mode 1, mode 2, mode 3, mode 13, mode 23, and mode 123. Mode 12 of the setting was rejected after preliminary tests because it returns low-quality results.

The dataset gathered from streams were captured using a GoPro Hero 6 camera (GoPro Inc., San Mateo, CA, USA), AXIS IP camera Q1765-LE (Axis Communications AB, Lund, Sweden), and Dahua IP camera IPC-HFW81230EZEH 12Mpx (Dahua Technology Co., Ltd., Hangzhou, China). Fig 4 contains five examples from dataset A. The size of each image is presented below each image.

The test datasets contain a representative sample of vessel images with varying quality with hull inscriptions of different sizes and placement, taken in variable lighting conditions. More information on data acquisition can be found in [36]. Two datasets, **A** and **B**, were used to test the quality and performance of the method working in different modes.

Dataset **A** contains 100 images (in bmp or png format) of vessels with hull inscription. The average file size is 646 KB. The dimensions of the images range from 269 x 524 to 1924 x 1670 pixels. The images were selected from our collection of ship images automatically cut from the video streams. Most of the picture area is covered by a ship. The images include whole ships and parts of ships, e.g., when a vessel enters the camera view. The collection contains images of 56 different ships. We selected several images of one ship taken at various intervals (ships with one image: 35; two images: 9, three images: 7, four images: 1, five images: 1, seven images: 1, eight images: 2). The images are from video streams from cameras with significant compression and have visible distortions associated with compression. We verified that a human could read the ship’s identifying inscriptions in all images.

Dataset **B** contains 100 images (in bmp or png format) of mostly vessels without hull inscriptions. The average file size is 391 KB. The dimensions of the images range from 104 x 100 to 1920 x 1066 pixels. This set is selected from the same cameras as set **A** but contains randomly selected images of ships, but only some have captions. In addition, several of the images do not include vessels.

In the experiments, three computers with the following specification were used:

1. Computer 1: Intel Core i7-8700K (Intel Corporation, Santa Clara, CA, USA), 32GB RAM, SSD 1TB, NVIDIA Quadro P4000, Windows 10 Pro.
2. Computer 2: Intel Core i7-8750H, 16GB RAM, SSD 256GB, NVIDIA GeForce GTX 1050Ti (Nvidia, Santa Clara, CA, USA), Microsoft Windows 10 Pro.
3. Computer 3: Intel Core i5-7440HQ, 16GB RAM, SSD 500GB, NVIDIA GeForce 930MX, Windows 10 Pro.

### Experiments

The purpose of the experiments was to investigate the speed and quality of vessels’ identification. Identification quality was measured in the percentage of correct results obtained in the dataset. We categorised the results into different types of matching. The ideal outcome is 100% full matches. First, the quality was tested using dataset A. After that, identification speed was measured in milliseconds on datasets A and B. The dataset B was also tested because the method construction causes images without titles to be processed longer.

**Fig 5:**
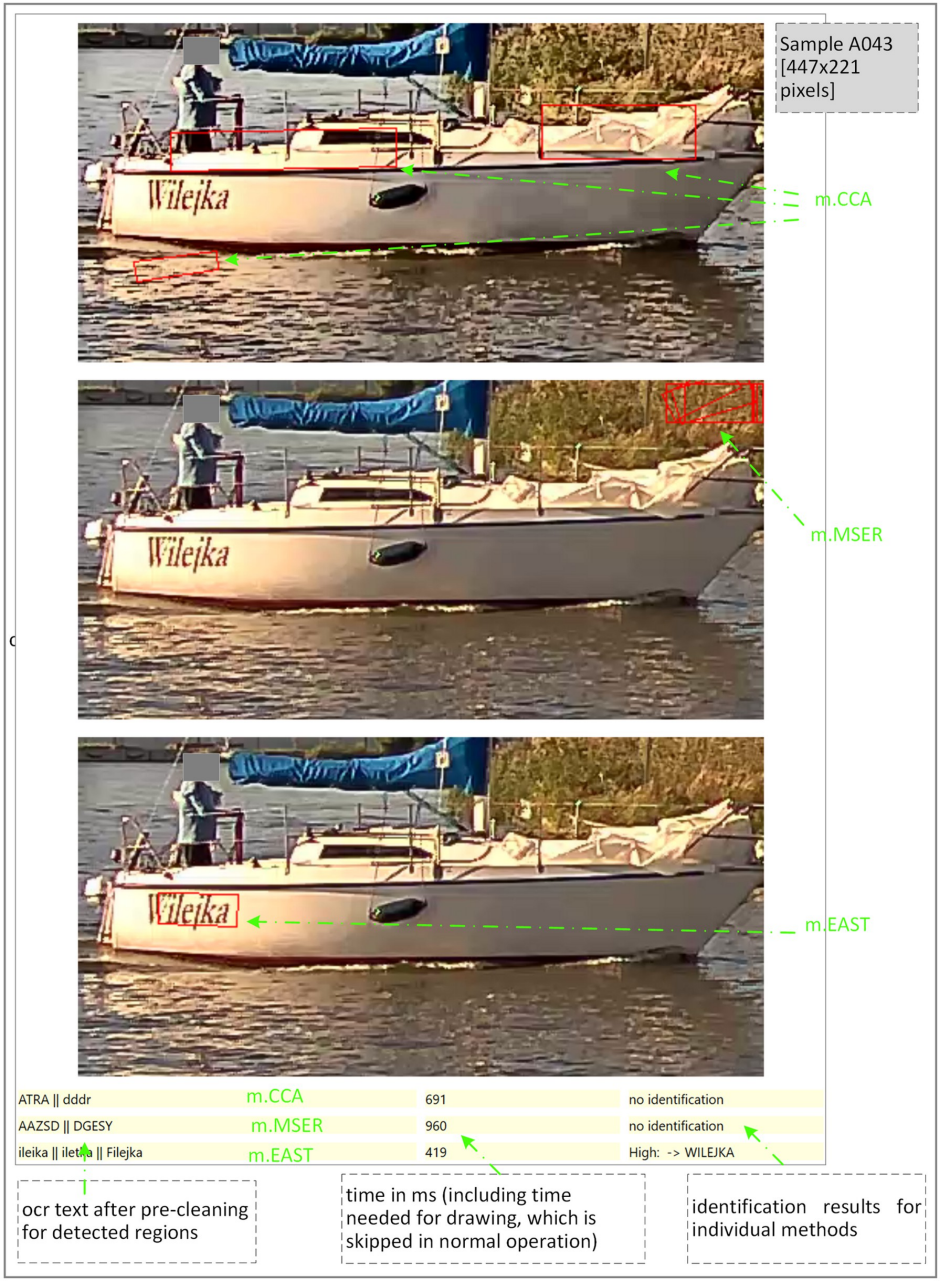
Example of text area localization by different methods in our data sets.

Before conducting the experiments, we conducted a series of preliminary tests that helped us to refine the proposed method. Fig 5 shows a labelled screenshot of the test application. It provides a visual analysis of the detection quality of the text areas, which are marked by a thin red frame.

**Fig 6:**
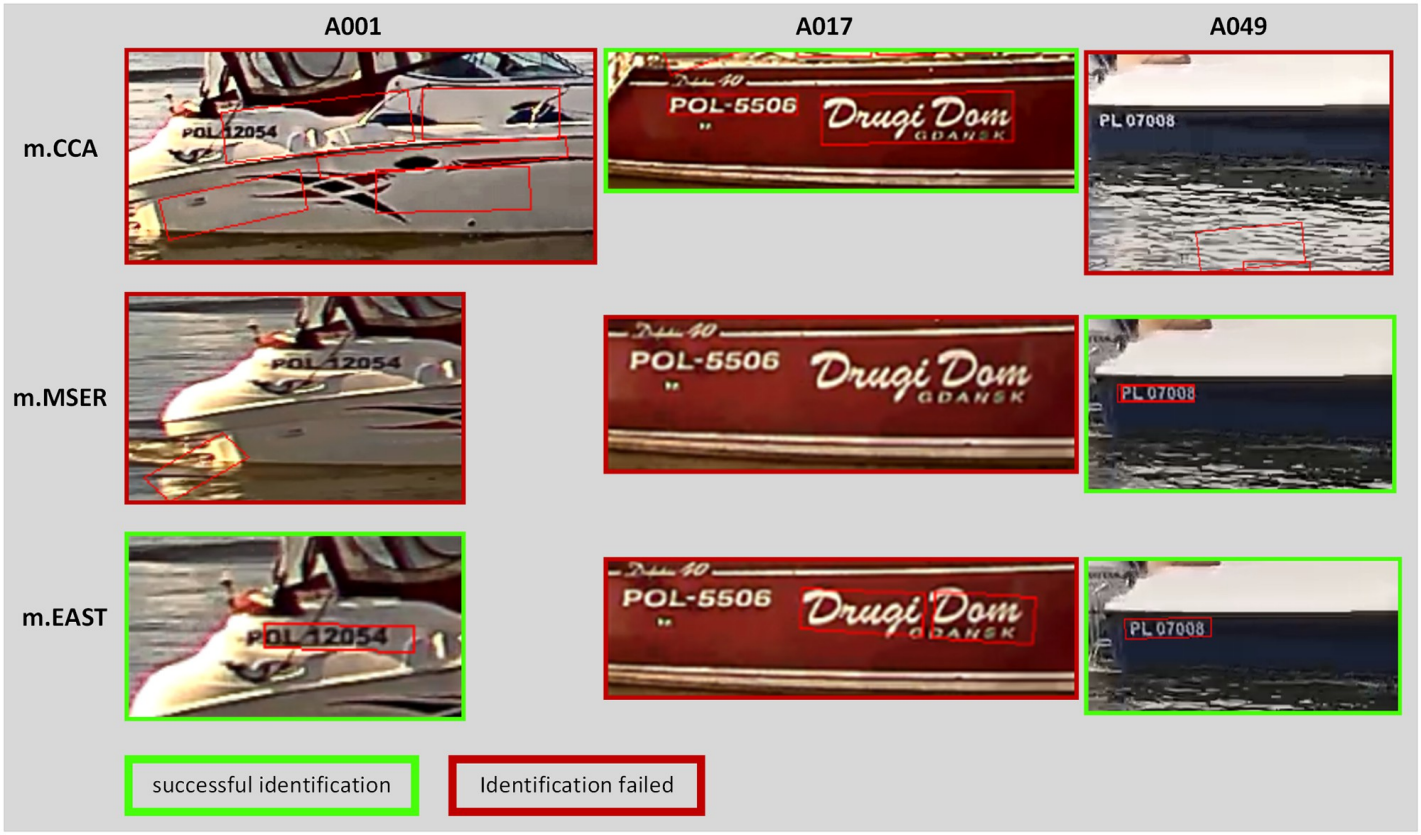
Examples of text localization in dataset A.

Fig 6 shows examples of the quality of the text location detection methods for three different samples from dataset A.

**Fig 7:**
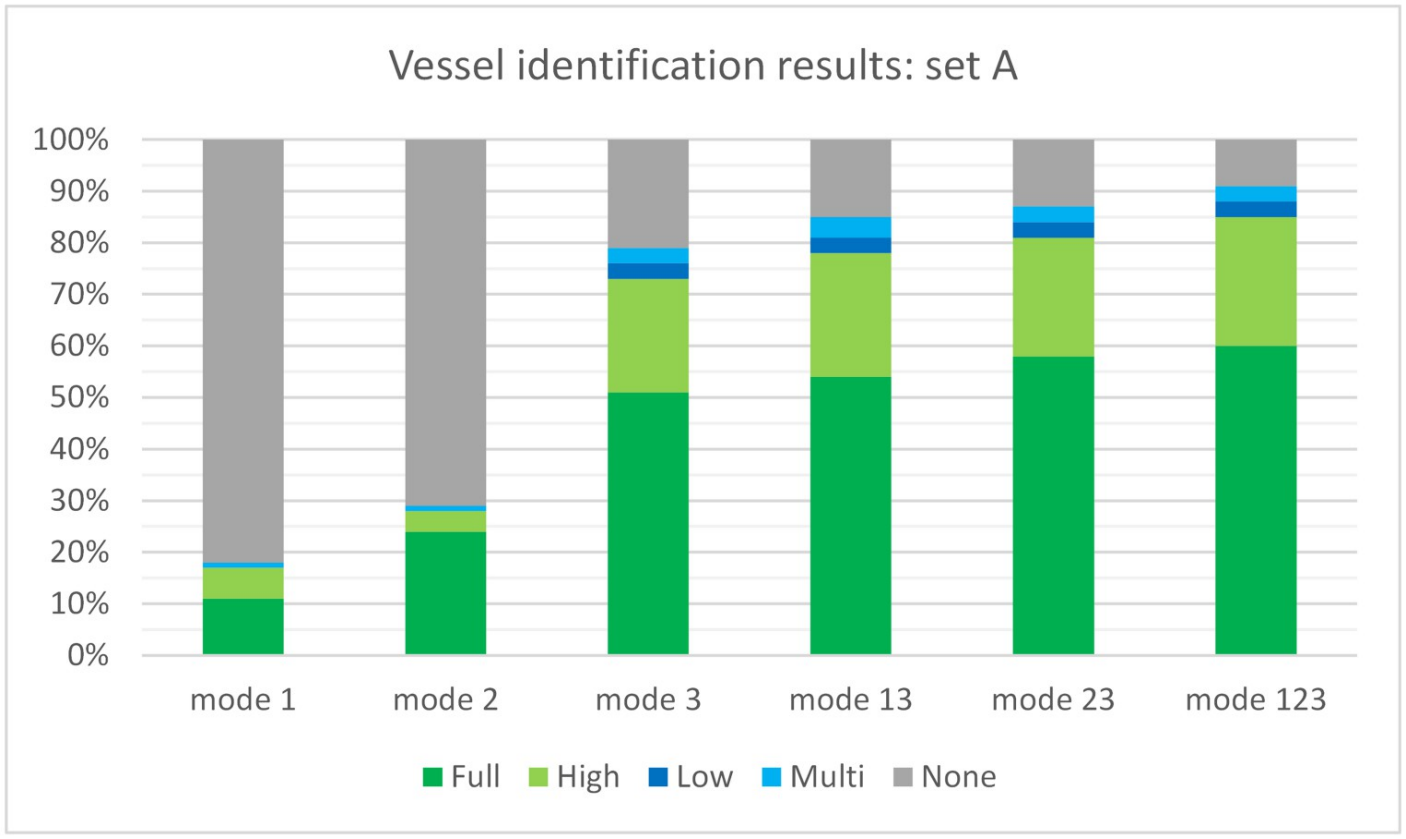
Identification quality for dataset A.

The identification quality of different modes of the method was tested using dataset A. The results are presented in Fig 7. The best mode that uses one scene text detection algorithm was the third mode (EAST) with identification quality equal to 79%, consisting of 51% full matches, 22% high matches, 3% low matches, and 3% multiple matches. The method working in modes 1(CCA) and 2 (MSER) returned rather low results, with mode 1 below 20% and mode 2 below 30%. Because modes 1–3 often identify different text areas, the results for combined modes are higher: 85% for mode 13, 87% for mode 23 (MSER+EAST), and 91% for mode 123 (CCA+MSER+EAST). They mostly consist of full matches and high matches with small number of low and multiple matches. The method working in combined mode 123 returned the best results, as expected, i.e., 60% full matches, 25% high matches, 3% low matches, 3% multiple matches, and 9% of vessels not identified.

The results of performance tests are shown in Tables 1 and 2 shows the speed of the method working in different modes. Mode 1 (based on Connected Component Analysis) is the fastest, with an average execution time between 268 and 367 ms depending on the test computer. The second is the third mode (based on the EAST Detector), with an execution time ranging from 317 to 567 ms. The slowest is the second mode, with detection based on Maximally Stable Extremal Regions (from 761 to 962 ms). The combined mode 13 execution time was between 389 and 660 ms, mode 23 between 505 and 811 ms, and mode 123 between 531 and 837 ms.

**Table 1:** Average vessel identification speed for dataset A.

Mode	Used method	Time [ms] Computer 1	Time [ms] Computer 2	Time [ms] Computer 3
Modes with single methods				
1	m.CCA	268	289	367
2	m.MSER	761	879	962
3	m.EAST	317	365	567
Mode with combined methods				
13	m.CCA+EAST	389	443	660
23	m.MSER+EAST	505	598	811
123	m.CCA+MSER+EAST	531	606	837

**Table 2:** Average vessel identification speed for comparative dataset B.

Mode	Used method	Time [ms] Computer 1	Time [ms] Computer 2	Time [ms] Computer 3
Modes with single methods				
1	m.CCA	218	235	293
2	m.MSER	458	533	592
3	m.EAST	243	285	479
Mode with combined methods				
13	m.CCA+EAST	449	517	770
23	m.MSER+EAST	695	821	1072
123	m.CCA+MSER+EAST	912	1041	1371

Table 2 presents results for dataset B, consisting mostly of images without inscriptions. The average execution time for the method working in modes 1–3 was lower than for dataset A (218 to 293 ms for mode 1, 458 to 592 ms for mode 2, and 243 to 479 ms for mode 3). This mainly results from the fact that fewer OCR operations were conducted (mostly on incorrectly detected titles’ areas). In comparison to Table 1, the execution times for the combined modes 13, 23, and 123 were significantly longer (449 to 720 ms for mode 13, 695 to 1072 for mode 23, and 912 to 1371 ms for mode 123). These times for combined modes are the sums of the execution times of the component modes, i.e., the execution time for a combined mode is the sum of the execution times for modes 1–3. This is since the method stops when a vessel is identified. When the inscription is absent, the methods iterate through all options.

The best time to quality ratio was found for the third mode (317 ms for dataset A and 243 ms for dataset B, using computer 1), with 79% of vessels identified on different match levels. To increase the quality to 91%, the execution time increases around 1.7-fold for images with inscriptions and 3.75-fold for images without inscriptions. Still, the maximum execution time on computer 1 was 912 ms, which allowed for an average identification rate of 1 per s.

## Discussion

Inland and coastal waterways are monitored using video cameras. Recently, the quality of video streams has improved and, in many places, full HD streams are used. In such cases, when a camera is placed on a bridge or a vessel is passing near the shoreline, it is possible to read different inscriptions. Of course, small vessels have smaller inscriptions that are more difficult to read than inscriptions of larger ships that are visible from a much greater distance. The commercial vessels generally have inscriptions placed according to regulations (i.e., regular fonts set against a contrasting background). The inscriptions on small pleasure vessels (especially in Poland) are made using different fonts, not necessarily with contrasting backgrounds, which makes them difficult to detect. Our method uses several scene text detection algorithms like this is the most challenging part because texts are sometimes tiny, i.e., the height of the letter is 10 pixels or fewer (10 pixels is our assumed threshold).

Another problem is that streams are usually highly compressed, which causes many disturbances in the image. In some cases, the letters are heavily disturbed, and it is impossible to recognise them. However, when one scene detection algorithm finds a text area correctly, the OCR engine (Tesseract OCR) usually returns partially correct results. The list of names and registration numbers of vessels (including pleasure ones) in Poland is public, so our method can match the text to names or registration numbers.

The matching algorithm is required as it is impossible to recognise inscription type accurately. Also, phone numbers are sometimes placed on vessels carrying tourists around the city. There are many registration numbers, and they often do not have a standard structure. Even when the structure is known, like an IMO number with seven digits (including one check digit), it is impossible to differentiate that number from a mobile phone number when we allow for matching with one error.

High matches are matches with one error, and low matches have two errors (not all types). The number of multiple matches in the experiments was low mainly because our list of vessels was limited and did not contain all vessels from vessel registries. The method is designed to be used in systems that constantly request identification and therefore multiple matches are useful when a series of images is analysed. In such cases, a correct identification can be deduced from several results. It should be noted that even a slight change of angle sometimes improves the OCR result and thereby improves the identification result. Additionally, the text must have a minimum of six signs to allow low matches with errors. Moreover, the constant prefixes of registration numbers are excluded to further minimize the possibility of a high number of multiple matches.

The vessels that were not identified by the method working in the combined mode (123) were not identified because the scene detection algorithms were not able to correctly find the text area. In future work, we plan to test newly developed methods. Currently, the method does not use any pre-processing (apart from deskewing) before using an OCR engine. We have tried many options (i.e., histogram equalization, increasing contrast, erosion), but they always produced worse results for our datasets. This might result from the fact that the text is very small and often not placed on a contrasting background.

The longest execution time was obtained for the method working in the second mode, which uses a scene detection algorithm based on Maximally Stable Extremal Regions (MSER). We have used MSER implementation built into EmguCV, which requires reading files with parameters every time text detection is requested and significantly impacts performance.

The method working in combined mode (123) sequentially calls detection methods and analyses their results. EAST Detector (mode 3) is used first. It returns a higher number of positive results. This allowed us to significantly reduce the execution time, as in most of the cases when the inscription is present, the other methods are not used. The execution time for the method is below 1 s, which is sufficient for practical purposes. However, there is room for improvement when a better scene text detection algorithm becomes available.

The proposed method differs significantly from existing methods of vessel identification using camera images, mainly due to its ability to recognize a wide range of vessels of many types using different hull marking rules. Ferreira et al. [37] proposed a solution for fishing vessels: first use a classifier to recognize fishing vessels’ prows and then find the text regions using a MSER algorithm. Authors admit that due to no standardisation of small ship markings the localization of text area and plate recognition is very problematic and give several examples of whole range of cases. In our case, there is not an easily distinguishable feature that would aid the process of text recognition for each type of vessel, although the classification of vessel types is a separate task in a SHREC project that can support the final decision on ship identification and has been covered in [38]. The method presented by Zhang et al. [33] also focused only on conventional (SOLAS) ships at the marine port of Dongying that have conventional, large, and distinct IMO hull markings, often locally accompanied by Chinese characters. Therefore, the authors enhanced the use of the EAST detector by adding FCN to properly detect horizontal text lines for Chinese characters. Authors have achieved text detection rate of 96.94 and text recognition rate of 93.54, with method’s average speed of 355 ms per single image. Huang et al. [34] presented an interesting end-to-end solution that includes solving the arbitrarily-oriented text issue but is also designed to work with clearly marked ships—naval warships including only large numbers. The recognition rate they achieved was 93.57 with the text area detection rate 95.16. Still this research concerned only large ships with standardized hull markings and their method achievement can be compared more to recognition process of car registration plates. To work somewhat universally, our method must use a hybrid approach to increase the chances of proper text recognition by OCR, because many NSTR algorithms give great results in some cases at the expense of others. Also, deep learning methods must be employed with caution because the system performance drops rapidly when processing high-resolution images.

In counteracting terrorism and fraud, our solution also has an advantage over the previously mentioned methods. None of these solutions uses ship registers to check the validity of the text recognition results. Although Zhang et al. used AIS Online information with timestamps on incoming ships, such information can be easily modified as AIS uses data manually entered into AIS transponders used on a vessel. Other methods do not involve any verification of recognised inscriptions.

## Conclusion

We have proposed a solution to the problem of recognizing poorly visible hull inscriptions from surveillance video, which is a subproblem of text recognition in natural scenes. Our solution, based on a combination of different text localization methods and additional processing and comparison of various character strings with existing ship identification data registers, allowed us to achieve a solution suitable for practical implementation. The processing time is sufficient for online processing, especially since the tests were performed specifically on mid-range, commonly available hardware. With the proposed approach, most of the ships can be identified, and the high-low matching subtitles (1, 2 errors) can be used when more vessel images are available.

The study’s main limitation is that the method requires access to a registry containing vessels data. When an unknown vessel appears in the monitored area, the method cannot identify it. However, the information or warning of a detected ship with a specific hull inscription can be passed, e.g., to a VTS/RIS operator. Also, the method is intended for administrative or governmental authorities that might be managing not only public registries.
